# Ugandan Men Exposed to Intimate Partner Violence: A Cross-Sectional Survey of Nationally Representative Data

**DOI:** 10.1007/s10935-022-00683-2

**Published:** 2022-06-01

**Authors:** Jacinta Waila, Herman Lule, Michael Lowery Wilson, Till Bärnighausen, Anne Abio

**Affiliations:** 1 Injury Epidemiology and Prevention (IEP) Research Group, Turku Brain Injury Centre, Turku University Hospital and University of Turku, Turku, Finland; 2 Department of Surgery, Directorate of Research and Innovations, Kampala International University, Kampala, Uganda; 3 Heidelberg Institute of Global Health (HIGH), University of Heidelberg, Heidelberg, Germany

**Keywords:** Violence, Community health, Mental health, Abuse, Domestic violence, Epidemiology, Sub Saharan Africa

## Abstract

Although women typically constitute the largest proportion of the population who experience the deleterious effects of intimate partner violence (IPV), understanding the bidirectional nature of IPV is important for developing nuanced prevention initiatives. This study examines data from the 2016 Ugandan Demographic and Health Survey. Participants were selected from households in all the 15 regions in Uganda using a two stage sampling design. A total of 2858 men who were in a heterosexual union or separated/divorced were included in the analysis. Univariate and multivariable logistic regression analyses were performed with the aim of identifying associations between selected demographic variables and male exposure to all forms of IPV combined, psychological violence, physical violence and sexual violence. The prevalence of lifetime IPV and during the 12 months preceeding the survey respectively was 43.6 and 30.5% in all forms, with 35.9 and 24.8% reporting psychological, 20.2 and 11.9% for physical and 8.2 and 5.7% sexual violence. The key factors associated with all forms of IPV were being afraid of their wife/partner most of the time (OR = 5.10, 95% CI 2.91, 8.96) controlling behaviour of the intimate partner (OR = 3.80, 95% CI 2.84, 5.07), bi-directional violence against the partner (OR = 3.20, 95% CI 2.49, 4.12), alcohol consumption by the intimate partner (OR = 1.85, 95% CI 1.40, 2.45). The factors associated with males who experience IPV appear to be modifiable and may warrant consideration for inclusion in programs supporting both males and females who experience IPV.

**Supplementary Information:**

The online version contains supplementary material available at 10.1007/s10935-022-00683-2.

## Introduction

Violence within intimate partnerships is a significant problem of public health importance worldwide. The WHO defines intimate partner violence (IPV) as ”any behaviour within an intimate relationship that causes physical, psychological or sexual harm to those in the relationship” (WHO, 2012). Contrary to the ubiquitous notion that IPV is almost always directed towards women, there is research highlighting women as perpetrators (Carmo et al., 2011; Hines  & Douglas, 2009; Hine et al., 2021; Park et al., 2021). Indeed, literature showing that women bear the greatest burden of IPV with regard to prevalence, severity of injuries and adverse consequences (Carbone-López et al., 2006; Tjaden  & Thoennes, 2000), has been challenged by research highlighting gender symmetry in IPV perpetration (Archer, 2000; Chen  & Chan, 2021). Furthermore, most violent incidents between intimate partners appear to be characterised by mutual aggression (Langhinrichsen-Rohling et al., 2012), sometimes initiated by the female partner (Powney  & Graham-Kevan, 2019). Consequently, despite trivialization of a relatively old argument that, within the family setting, women are just as violent as men (Straus  & Gelles, 1986), coupled with a global focus on women as the main targets of IPV, this proposition seems to be gaining support.

The WHO global estimates show that 30% of women who have been in an intimate relationship have experienced IPV, a figure which stands at 36.6% in the African region (WHO, 2013). Similar estimates for male IPV are unavailable but several scholars have been able to estimate the IPV prevalence in various world regions. For instance, nationally representative data reveal the lifetime prevalence of any form of male IPV to be 22.9% (Coker et al., 2002), with physical IPV estimated at about 15% in the United States (Breiding, 2014; Breiding et al., 2008). In Europe, lifetime psychological, sexual and physical IPV prevalence among men is estimated to be as high as 72%, 27% and 31% in some cities (Costa et al., 2015). In the East African region, a nationally representative sample in Kenya estimated the lifetime experience of any form of IPV among ever partnered men between the ages of 15 and 49 years to be 24% with the most reported form of IPV being psychological at 21% and sexual violence the lowest at 4% (KNBS et al., 2015). In Dar es Salaam, Tanzania 34.8% of men reported experiencing any form of IPV within the preceding 12 months (Mulawa et al., 2016). Death is undeniably the worst but not the only adverse outcome of IPV often affecting intimate partners but also other individuals such as their children, friends and relatives (Smith et al., 2014).

Psychological IPV is more strongly associated with negative physical and mental health outcomes than physical IPV (Coker et al., 2002). Post-traumatic stress disorder (PTSD), alcohol and substance abuse, anxiety and depression are some of the mental disorders linked to male IPV experience (Coker et al., 2002; Carbone-López et al., 2006; Hines  & Douglas, 2009; Lagdon et al., 2014). Further, chronic disorders such as hypertension and diabetes mellitus are also associated with male IPV (Coker et al., 2002). Though physical injuries may not be severe when IPV is directed towards men, women, especially when using harm-inflicting objects or weapons, have the potential to cause serious injuries (Busch  & Rosenberg, 2004; Hines  & Douglas, 2009). Abrasions, broken bones, tooth loss, injury to sensory organs, burns as well as stab and gunshot wounds are some of the documented physical injuries inflicted on men (Carbone-López et al., 2006; Carmo et al., 2011; Hines  & Douglas, 2009; Tjaden  & Thoennes, 2000). In spite of these serious adverse health outcomes, male IPV is still under-reported due to the perception that such violence does not conform to societal norms, a notion that attracts ridicule and shame (Bates, 2020; Douglas  & Hines, 2011).

Besides health consequences, the economic losses occasioned by IPV are significant, most of which are related to lost productivity, medical bills, property loss or damage and criminal justice costs (King et al., 2017; Peterson et al., 2018). Being relatively young, unemployed or having a low income, low educational attainment, belonging to certain ethnic groups and residing in certain regions are some of the documented socio-economic factors associated with IPV experience among men (Breiding et al., 2008; Cunradi et al., 2002; Marie et al., 2008; Mulawa et al., 2016). Similarly, alcohol and substance abuse, antisocial personality and being from a dysfunctional family environment where one is exposed to parental alcoholism, illicit drug use, interpersonal violence and childhood exposure to abuse are developmental and behavioural factors closely linked to male IPV (Carmo et al., 2011; Linder  & Collins, 2005; Marie et al., 2008; White  & Widom, 2003). Partner dominance often manifested through controlling behaviours is commonly linked to relationship dissatisfaction, a substantial risk factor for IPV experience not just for men but women alike (Bates, 2016; Slep et al., 2010).

Though some scholars posit that women bear the burden of IPV disproportionately, the availability of literature highlighting gender symmetry in IPV perpetration and similar consequences in men and women is a call for deliberate exploration of male IPV. The bidirectional nature of most IPV occurrences calls for a holistic understanding of the problem if prevention strategies are to be effective (Bates, 2016; Hines  & Douglas, 2009; Langhinrichsen-Rohling et al., 2012). The adoption of this holistic approach necessitates deliberate efforts in generating knowledge on male IPV, and more especially in sub Saharan Africa countries which despite reporting high prevalence of IPV directed towards women (Devries et al., 2013; WHO, 2013), have data evidence on male experienced IPV. Nationally representative data from Uganda, a low-income country in East Africa, shows that about 56% of married or cohabiting women between the ages of 15 and 49 years have experienced at least one form of IPV with physical IPV being the most reported type at 41% (Gubi et al., 2020). While some of the IPV prevention interventions in Uganda focus on social behaviour change communication to influence the adoption of tolerant social norms and behaviours (Ashburn et al., 2017; Michaels-Igbokwe et al., 2016; Wagman et al., 2012), a glaring gap in the fight against IPV is the mere assumption that men are the perpetrators yet male IPV in the country has not been characterised. Using nationally representative Uganda Demographic Health Survey 2016 data, this study seeks to describe male IPV in Uganda by estimating its burden and associated factors.

## Methods

### Data and Population

The data were derived from the 2016 Ugandan contribution to the Demographic Health Survey (DHS). The DHS is a cross-sectional survey and the data are collected from a nationally representative sample of the entire population. The survey was conducted by the Uganda Bureau of Statistics (UBOS). A two-stage stratified cluster sampling design was used to select the population for participation. All the 15 regions that were available from the 2014 National Population and Housing Census were represented in the survey. Moreover, the survey used Enumeration Areas (EAs) that were created during the census. A total of 697 EAs were selected in the first stage (162 EAs in urban areas and 535 in rural areas), while the second stage involved a selection of households within an EA or a segment of the EA if it contained more than 300 households. A total of 30 households per EA or segment from an EA were randomly selected resulting in a total of 20 880 households. Males aged 15 to 54 years from one third of the sampled households (one per selected household) were eligible to participate in the survey. Data collection took place from 20 June 2016 to 16 December 2016. The data derived from this survey are publicly available. Further details about the survey, methodology and ethical approval are available elsewhere (ICF. The DHS Program - Uganda: Standard DHS. Funded by USAID, 2016).

### Participants

Out of a total of 5 676 eligible men selected from households, 5 336 were successfully interviewed. A total of 4 011 males responded to the 2016 domestic violence module of the Ugandan DHS while 2 858 male participants responded to the IPV questions used in this study and were included in the analysis. The respondents were either in a heterosexual union (married or cohabiting) or separated/divorced.

### Variables of Interest

The outcome of interest was the reported experience of IPV among males. IPV experience was further subdivided into psychological, physical and sexual violence based on three of the four major typologies of violence as described by WHO (Krug et al., 2002). In this case, a binary variable about IPV was created, based on if the participants reported having ever experienced any of the three types of violence. The questions on psychological violence included whether their wives/partners had ever humiliated or threatened them. The questions on physical violence included whether the respondents had ever been *hit, kicked, slapped, punched, pushed, threatened, strangled or burnt* by their wives/partners. Moreover, the questions on sexual violence included if the respondents had been *forced to have unwanted sex or perform sexual acts with their wives/partners*. The associated exploratory factors investigated in the study included age, sex, place of residence (rural vs urban), employment, income, the level of education, number of children, smoking status, number of sexual partners, whether they had given gifts for sex, wife drank alcohol, bi-directional violence and controlling behaviour (Supplementary Table 1).

### Statistical Analysis

Descriptive statistics were calculated using based svy Pearson Chi-squared tests. Logistic regression analysis was used to determine the strength and direction of associations between the outcomes of interest and the associated covariates. Univariate and multivariable logistic regression was used to determine associations between each individual covariate. Variables were retained in a multivariable model based on *p* = 0.1. The statistical significance used was 0.05 with a 95% confidence interval. The survey-based design was used both for Chi-squared tests and the logistic regression analysis. The svylogitgof (Archer  & Lemeshow, 2006) was used to test for the fit of the multivariable models, and were found to have a good fit. Stata 17 (StataCorp, TX, USA) was used for the analysis.

## Results

A total of 1 264 (43.6%) and 905 (30.5%) of the males in this study had ever experienced any form of IPV during their lifetime and over the last 12 months respectively (Table 1). Among the three types of violence, 35.9% and 24.8% reported having experienced psychological violence, 20.2% and 11.9% physical violence and 8.2% and 5.7% sexual violence within the recall periods over their lifetime and the last 12 months respectively. A majority of the respondents reported being currently married or cohabiting (91.4%), and living in rural areas (78.0%) at the time of the survey (Table 2).

The study found that 21.1% of the respondents were afraid of their spouses sometimes or most of the time, while 40.7% had witnessed parental abuse (father beat mother). Moreover, 22% reported hurting their wife/partner when she was not hurting him signifying bi-directional violence, and 77.7% had experienced controlling behaviour from their partners. In the unadjusted logistic models (Table 3), the factors associated with all forms of IPV were being afraid of the wife most of the time (OR 7.94, 95% CI 4.39, 14.37), controlling behaviour (OR 4.76, 95% CI 3.59, 6.29) and hurting the wife when she was not hurting him (OR 4.39, 95% CI 3.47, 5.56). Males who had a wife or wives were less likely to experience IPV. On the other hand, the respondents who were separated or divorced were twice as likely to experience IPV compared to those currently in a union. Similar associations were observed for psychological violence with being afraid most of the time (OR 5.89, 95% CI 3.21, 10.80), controlling behaviour (OR 6.26, 95% CI 4.57, 8.59) and hurt wife/partner (OR 4.10, 95% CI 3.25, 5.16).

On the other hand, the factors associated with physical violence were being afraid of the wife/partner most of the time (OR 7.37, 95% CI 4.24, 12.79), wife/partner often being drunk (OR 6.31, 95% CI 3.00, 13.28) and bi-directional violence against the wife/partner (OR 4.00, 95% CI 3.19, 5.03). Sexual violence was strongly associated with controlling behaviour (OR 7.53, 95% CI 4.20, 13.49), wife/partner often being drunk (OR 6.09, 95% CI 1.99, 18.63) and being afraid of the wife/partner most of the time (OR 4.62, 95% CI 2.45, 8.71).

Congruently, in the multivariable models (Table 4), the factors associated with all forms of IPV, were being afraid of the wife/partner most of the time (OR 5.10, 95% CI 2.91, 8.96), controlling behaviour (OR 3.80, 95% CI 2.84, 5.07), and bi-direction violence against the wife/partner (OR 3.20, 95% CI 2.49, 4.12). Similar factors were observed for psychological violence, with controlling behaviour (OR 4.93, 95% CI 3.57, 6.81), being afraid of the wife/partner most of the time (OR 4.28, 95% CI 2.50, 7.31) and bi-directional violence (OR 2.99, 95% CI 2.33, 3.84). Employed respondents were more likely to experience psychological violence (OR 2.70, 95% CI 1.29, 5.66), and those between 35−44 years (OR 1.77, 95% CI 1.26, 2.47).

The factors associated with physical violence were being afraid most of the time (OR 4.99, 95% CI 2.91, 8.53), bi-directional violence (OR 2.99, 95%2.36, 3.78) and the wife/partner drinking alcohol (OR 2.29, 95% CI 1.74, 3.01). On the other hand, sexual violence was associated with controlling behaviour (OR 5.73, 95% CI 3.14, 10.46), and being afraid of the wife most of the time (OR 3.59, 95% CI 1.87, 6.91). Respondents from the higher wealth indices were also more likely to experience sexual violence compared to the poorest index. Additionally, the age group (35−44) were less likely to experience sexual violence compared to those 15−24 years of age.

## Discussion

Overall, the lifetime prevalence of all forms of IPV among males in Uganda was 44% and 31% during the 12 months preceding the survey. The factors associated with all forms of IPV included being afraid of their partners, controlling behaviour from the partners and bi-directional violence by hurting the partner when she was not hurting him. IPV has been more commonly studied among females compared to males. Based on the high prevalence which - close to half of the selected male respondents, it is evident that this a subject that is less frequently addressed among males.

The lifetime prevalence of IPV was higher than that reported in Tanzania and the US ranging from 7 to 34.8% (Coker et al., 2002; Mulawa et al. 2016; Tjaden  & Thoennes 2000). The high rates could be attributed to the high prevalence of witnessing parental violence and controlling behaviour. In this study, approximately, 40% of the males had witnessed parental violence while in other studies the estimate has ranged from 36 to 52% (Gubi et al. 2020; Kwagala et al. 2013). Moreover, approximately three quarters of the selected respondents had experienced controlling behaviour from their partners. A combination of exposure to these factors may have influenced the higher prevalence of IPV observed.

Regarding the three forms of violence, psychological violence had the highest prevalence which is consistent with other studies (Costa et al., 2015; Ferraresso, 2020). Rates of psychological violence were also higher than reported in studies from Europe, the United States and South Korea (Coker et al., 2002; Costa et al., 2015; Ferraresso, 2020). Psychological violence may be higher among males as the female perpetrators are more likely to report psychological aggression and may be less inclined to inflict bodily harm and injuries on their intimate partners (Carmo et al., 2011; Karakurt  & Silver, 2013). Furthermore, due to prevailing societal and gender norms, it is more socially acceptable for females to engage in verbal aggression rather than physical aggression (Karakurt  & Silver, 2013).

Physical violence was the second most prevalent form of IPV which was higher than rates observed in Europe, (except Athens at 31.2%), United States and South Korea (Coker et al., 2002; Costa et al., 2015; Ferraresso, 2020). This is however inconsistent with a study from Tanzania that found sexual violence as the second most prevalent form of IPV among males at 11.1%, after psychological violence (Mulawa et al., 2016). The difference could be due to the study settings. The Tanzania study was conducted in Dar es Salaam which is largest city in the country and thus represented the prevalence in only an urban setting. This study used data that was derived from a national representative sample of the entire country, whose survey population is largely rural.

Bidirectional violence was a major risk factor for IPV as reported in other studies (Costa et al., 2015; Ferraresso, 2020; Hamel  & Russell, 2012; Powney  & Graham-Kevan, 2019). For example, more than half of the selected study population who experienced IPV and psychological violence (71% and 62% respectively) admitted to perpetrating violence against their partners. This is higher than reported in a review of studies (49–57.9%) (Hamel  & Russell, 2012; Powney  & Graham-Kevan, 2019) which is a high prevalence nonetheless. A number of male victims may equally be perpetrators of IPV within their settings (Bates, 2016; Coker et al., 2002; Mulawa et al., 2016). The implication is that preventive efforts to tackle IPV may need to target couples to break the cycle of violence as the victims may equally have been perpetrators.

Being afraid of their respective partners was also associated with IPV as documented in the United Kingdom (Taylor et al., 2021). This has also been reported as a factor in studies conducted among females (Kwagala et al., 2013; Wandera et al., 2015). Fear of partners is normally associated with past behaviours from a partner that could have arisen through threats or fear of bodily injuries in order to exert power or control in a relationship. Men who have expressed fear of their partners are less likely to seek help (Powney  & Graham-Kevan, 2019; Taylor et al., 2021). The factors associated with fear in men include being scared of retaliation, fear of the female abusers falsely claiming to be the victim to law enforcement or peers, fear of ultimately being rejected by their family and children as well as fear for their well-being (Taylor et al., 2021). Although it is anticipated that men may not be afraid of their partners, this may not entirely be the case considering the percentage of men who were afraid of their partners in our study.

Controlling behaviour from the males’ partners was also associated with all forms of IPV as reported in other studies (Ferraresso, 2020; Gubi et al., 2020). Controlling behaviour constitutes but is not limited to threats, intimidation, destruction of property, isolation of victims, exerting control over finances and legal abuse (Ferraresso, 2020; Powney et al., 2021). Controlling behaviour can be harmful to the victims as it is associated with mental distress, alcohol use, substance abuse and reduced self-confidence (Powney et al., 2021).

The age group 25-34 years had a lower odds of experiencing sexual violence compared to those younger than 25, which is similar to a study conducted among females in Uganda (Gubi et al., 2020). The older age groups 35-54 were also less likely to experience sexual violence although it was not statistically significant. With the more recent exposure to social media and digital technology, sexual communication has become more common among young adults. Drouin et al. found that sexual coercion was associated with sexual violence and similar proportions were observed among males and females (Drouin et al., 2015). The sexual coercion involved making respondents feel obligated or due to persistent requests to go ahead with unwanted things (Drouin et al., 2015). Given the increasing use of technology, a preventive measure to reduce the occurrence of sexual violence among the younger generation would require strengthened and comprehensive sex education targeting both boys and girls to minimise perpetration and improve assertiveness for the potential victims.

All the wealthier respondents were more likely to report sexual IPV compared to the poorest in our study. Considering that wealthier people are more likely to access health information (Tang et al., 2019), it is possible that they are more enlightened about IPV and are therefore confident to acknowledge their experiences with it compared to their less empowered counterparts. Based on this, our findings could be more reflective of a reporting pattern than the actual experience of male IPV in our study population.

Other factors that have typically been associated with IPV among females were not associated with male IPV in this study. For example, the level of education was not associated with any form of IPV. This is similar to a study among males in which education was not associated with IPV in Tanzania and South Korea (Ferraresso, 2020; Mulawa et al., 2016), while males with incomplete primary education in Rwanda were more likely to experience psychological violence (Umubyeyi et al., 2014). Women with lower education attainment are more likely to report IPV in studies from Uganda and Rwanda (Gubi et al., 2020; Umubyeyi et al., 2014). Males may be less inclined to report IPV experiences due to fear of ridicule and cultural norms that expect them to be brave (Umubyeyi et al., 2014).

In our study, males who were employed had a higher risk of psychological violence which was consistent with a South Korean study (Ferraresso, 2020). However, females with professional employment status had a lower risk of physical violence in another Ugandan study (Kwagala et al., 2013). The number of children has been associated with IPV among females (Gubi et al., 2020; Kwagala et al., 2013) but not in our study. It is considered more of a woman’s responsibility to look after children within the sub Saharan African context. Therefore, the they are likely to remain in an abusive relationship for the sake of the children. Thus, the risk factors of violence among females may necessarily not be the same among males.

### Strengths and limitations

Strengths: The study contributes significant knowledge on intimate violence among males in sub Saharan Africa. There is a paucity of information on the subject among males as it tends to get overlooked in comparison to females. While we acknowledge that there is an imbalance in terms of limited access to resources among females which predisposes them to IPV, the prevalence rates in this study indicate that males are also predisposed to violence. Another strength is that the study was conducted among a nationally representative sample of the Ugandan population, thus the rates are generalisable to the country. Limitations: Considering that IPV among males is often stigmatised, denied or ridiculed and could be under-reported due to cultural norms (Carmo et al., 2011; Karakurt  & Silver, 2013; Umubyeyi et al., 2014), it is possible that the estimate presented may be conservative and influenced by reporting bias. Secondly, the cross-sectional survey by design is unable to establish causality, therefore it was not possible to determine the factors that caused the violence. Thirdly, the questions used in this study about the experience of IPV were asked from persons who were currently or formerly in a union but not from those who had never married or had not lived together as a couple. Spousal violence is not only limited to couples who live together. Therefore the prevalence of IPV within males who had never lived together as a couple was not estimated in this study, although it is possible this demographic has experienced violence through dating and it is important to have their experiences captured.

## Conclusion

Two out of five males have experienced IPV in Uganda. Major factors include feeling afraid of the partner, perpetrating the violence and exposure to controlling behaviour from the partner. Preventive measures aimed at addressing IPV at the community level may benefit by targeting couples and the inclusion of men in IPV prevention initiatives. Further research on IPV experience among men may be warranted given the high numbers of men who experience it and who appear to be less likely to seek help.

**Table 1:** Descriptive statistics of intimate partner violence against males in Uganda (2016) – unweighted numbers and weighted percent over lifetime and past 12 months

Variable	IPV (lifetime)	No IPV (lifetime)	IPV (12 mo.	No IPV (12 mo.)
*IPV (all types)*	1264 (43.6)	1594 (56.4)	905 (30.5)	1954 (69.5)
Psychological violence	1053 (35.9)	1805 (64.1)	750 (24.8)	2108 (75.2)
Physical violence	579 (20.2)	2279 (79.8)	348 (11.9)	2510 (88.2)
Sexual violence	237 (8.2)	2621 (91.8)	161 (5.7)	2697 (94.3)
*Age*				
15–24	156 (43.3)	201 (56.7)	132 (36.9)	225 (63.1)
25–34	505 (43.0)	657 (57.0)	395 (34.0)	767 (66.0)
35–44	381 (46.1)	439 (53.9)	245 (28.6)	575 (71.4)
45–54	222 (41.4)	297 (58.6)	132 (23.2)	387 (76.8)
*Place of residence*				
Rural	1081 (43.8)	1279 (56.2)	722 (30.4)	1575 (69.6)
Urban	246 (42.9)	315 (57.1)	182 (30.8)	379 (69.2)
*Level of education*				
No education	69 (39.8)	91 (60.2)	50 (27.4)	110 (72.6)
Primary	769 (45.8)	914 (54.2)	539 (31.0)	1144 (69.0)
Secondary	275 (41.4)	382 (58.6)	203 (30.4)	454 (69.6)
Tertiary	151 (39.8)	207 (60.2)	112 (29.7)	246 (70.3)
*Marital status*				
Currently married/union	1119 (41.8)	1506 (58.2)	839 (30.8)	1786 (69.2)
Separated/divorced	145 (62.6)	88 (37.4)	65 (27.0)	168 (73.0)
*Employment*				
No	26 (47.4)	25 (52.6)	19 (34.7)	32 (65.3)
Yes	1238 (43.6)	1569 (56.4)	885 (30.4)	1922 (69.6)
*Wealth index*				
Poorest	274 (41.2)	381 (58.8)	194 (28.1)	461 (71.9)
Poorer	281 (44.4)	316 (55.6)	209 (33.6)	388 (66.7)
Middle	258 (45.9)	308 (54.1)	186 (32.5)	380 (67.5)
Richer	249 (45.0)	304 (55.0)	176 (29.9)	377 (70.1)
Richest	202 (41.4)	285 (58.6)	139 (28.5)	348 (71.5)
*Number of children*				
0	64 (42.7)	99 (57.3)	49 (34.3)	114 (65.7)
1–4	591 (42.6)	799 (57.4)	446 (31.8)	944 (68.2)
5+	609 (44.8)	696 (55.2)	409 (28.7)	896 (71.3)
*Smoking*				
Does not smoke	1037 (41.3)	1419 (58.7)	756 (29.7)	1700 (70.3)
Some days	50 (53.6)	44 (46.4)	37 (38.6)	57 (61.4)
Everyday	177 (59.7)	131 (40.3)	111 (34.0)	197 (66.0)
*Number of wives*				
0 (Separated/Divorced)	145 (62.6)	88 (37.4)	65 (27.0)	168 (73.0)
1	919 (41.3)	1296 (58.7)	685 (30.3)	1530 (69.7)
2+	200 (45.3)	210 (54.7)	154 (33.9)	256 (66.1)
*No. sex partners (past 12 mo.)*				
None	894 (39.6)	1354 (60.4)	635 (27.4)	1613 (72.6)
One or more	370 (58.8)	240 (41.2)	269 (42.2)	341 (57.8)
*Given gifts for sex (past 12 mo.)*				
No	1165 (42.4)	1546 (57.6)	833 (29.7)	1878 (70.3)
Yes	99 (65.5)	47 (34.5)	71 (45.3)	75 (54.7)
*Respondent afraid of wife*				
Never	855 (37.3)	1546 (62.7)	592 (25.1)	1661 (74.9)
Sometimes	342 (65.2)	178 (34.8)	258 (48.3)	262 (32.3)
Most of the time	67 (82.5)	18 (17.5)	54 (67.7)	31 (51.7)
*Father beat mother*				
No	573 (38.0)	947 (62.0)	406 (26.4)	1114 (73.6)
Yes	605 (50.1)	554 (49.9)	438 (35.5)	721 (64.5)
Don’t know	86 (50.0)	93 (50.0)	60 (33.4)	119 (66.6)
*Wife drinks alcohol*				
No	913 (39.3)	1379 (60.7)	655 (27.6)	1637 (72.4)
Yes	351 (62.5)	215 (37.5)	249 (43.0)	317 (57.0)
*Frequency of wife being drunk*				
Never	74 (51.4)	66 (48.6)	50 (32.8)	90 (67.2)
Sometimes	218 (63.9)	135 (36.1)	157 (45.3)	196 (54.7)
Often	59 (79.9)	14 (20.1)	42 (53.9)	31 (46.1)
Bi-directional violence*				
No	809 (35.9)	1413 (64.1)	586 (25.5)	1636 (74.5)
Yes	455 (71.1)	181 (28.9)	318 (48.1)	318 (51.9)
*Controlling behaviour*				
No	116 (18.2)	505 (81.8)	73 (10.1)	548 (89.9)
Yes	1148 (51.1)	1089 (48.9)	831 (36.5)	1406 (63.5)

12 mo. refers to IPV experience in the 12 months preceding the survey

*Hurt wife/partner when she was not hurting the male respondent

**Table 2:** Cross-tabulations of males who reported experiencing intimate partner violence in Uganda (2016)

Variable	All IPV	*p*	Psychological	*p*	Physical	*p*	Sexual	*p*
*Age*		0.530		0.022		0.390		0.059
15–24	156 (43.3)		119 (31.1)		65 (19.4)		44 (11.5)	
25–34	505 (43.0)		417 (35.7)		238 (20.0)		102 (9.3)	
35–44	381 (46.1)		334 (40.5)		157 (18.7)		53 (6.5)	
45–54	222 (41.4)		183 (32.5)		119 (23.3)		38 (6.7)	
*Place of residence*		0.763		0.847		0.360		0.546
Rural	1081 (43.8)		837 (35.8)		475 (20.7)		182 (8.0)	
Urban	246 (42.9)		216 (36.4)		104 (18.5)		55 (9.0)	
*Level of education*		0.255		0.241		0.030		0.285
No education	69 (39.8)		53 (34.4)		38 (22.6)		12 (6.5)	
Primary	769 (45.8)		654 (38.0)		365 (22.4)		136 (8.5)	
Secondary	275 (41.4)		220 (32.1)		112 (17.0)		62 (9.3)	
Higher	151 (39.8)		126 (34.4)		64 (15.5)		27 (5.6)	
*Marital status*		< 0.001		< 0.001		< 0.001		0.002
Currently married/union	1119 (41.8)		924 (34.0)		498 (18.9)		193 (7.6)	
Separated/divorced	145 (62.6)		129 (56.6)		81 (34.1)		44 (14.4)	
*Employment*		0.653		0.270		0.232		0.983
No	26 (47.4)		18 (27.7)		16 (28.3)		5 (8.3)	
Yes	1238 (43.6)		1035 (36.0)		563 (20.1)		232 (8.2)	
*Wealth index*		0.634		0.687		0.311		0.158
Poorest	274 (41.2)		229 (34.2)		124 (18.3)		35 (5.0)	
Poorer	281 (44.4)		228 (34.4)		144 (23.6)		58 (10.0)	
Middle	258 (45.9)		215 (38.4)		118 (20.9)		47 (8.4)	
Richer	249 (45.0)		211 (37.4)		106 (20.7)		51 (8.9)	
Richest	202 (41.4)		170 (34.8)		87 (17.6)		46 (8.6)	
*No. of children*		0.624		0.283		0.250		0.189
0	64 (42.7)		46 (28.9)		30 (23.0)		17 (9.8)	
1	591 (42.6)		490 (35.8)		259 (18.4)		128 (9.2)	
5+	609 (44.8)		517 (36.9)		290 (21.7)		92 (7.0)	
*Smoking*		< 0.001		0.0001		0.0001		0.265
Does not smoke	1037 (41.3)		861 (33.9)		464 (18.4)		194 (8.0)	
Some days	50 (53.6)		44 (45.5)		27 (32.1)		10 (6.7)	
Everyday	177 (59.7)		148 (49.8)		88 (31.5)		92 (10.8)	
*Number of wives*		< 0.001		< 0.001		< 0.001		0.008
0 (Divorced/Separated)	145 (62.6)		129 (56.6)		81 (34.1)		44 (14.4)	
1	919 (41.3)		762 (33.4)		402 (18.3)		162 (7.8)	
2+	200 (45.3)		162 (37.1)		96 (22.6)		31 (6.9)	
*No. of sex partners*		< 0.001		< 0.001		< 0.001		0.0003
None	894 (39.6)		738 (32.2)		404 (17.9)		148 (7.0)	
One or more	370 (58.8)		315 (50.0)		175 (29.1)		89 (12.7)	
*Given gifts for sex*		< 0.001		< 0.001		< 0.001		0.0001
No	1165 (42.4)		970 (34.9)		528 (19.3)		207 (7.7)	
Yes	99 (65.5)		83 (55.5)		51 (38.3)		27 (18.4)	
*Afraid of wife*		< 0.001		< 0.001		< 0.001		< 0.001
Never	855 (37.3)		706 (30.7)		340 (15.3)		146 (6.2)	
Sometimes	342 (65.2)		288 (53.0)		191 (36.3)		74 (14.5)	
Most of the time	67 (82.5)		59 (72.3)		48 (57.0)		17 (23.5)	
*Father beat mother*		< 0.001		< 0.001		0.0004		0.341
No	573 (38.0)		468 (31.0)		256 (17.1)		103 (7.4)	
Yes	605 (50.1)		513 (41.4)		269 (23.0)		117 (9.3)	
Don’t know	86 (50.0)		72 (42.3)		54 (29.6)		17 (8.2)	
*Wife drinks alcohol*		< 0.001		< 0.001		< 0.001		0.581
No	913 (39.3)		750 (31.8)		371 (16.3)		180 (8.1)	
Yes	351 (62.5)		303 (54.0)		208 (37.6)		57 (8.9)	
*Frequency of wife being drunk*		0.003		0.147		< 0.001		0.006
Never	74 (51.4)		70 (48.3)		25 (19.0)		6 (3.1)	
Sometimes	218 (63.9)		181 (54.0)		140 (41.3)		38 (9.9)	
Often	59 (79.9)		52 (66.0)		43 (59.7)		13 (16.5)	
Bi-directional violence*		< 0.001		< 0.001		< 0.001		< 0.001
No	809 (35.9)		657 (28.6)		327 (14.5)		145 (6.6)	
Yes	455 (71.1)		396 (62.1)		252 (40.5)		92 (13.9)	
*Controlling behaviour*		< 0.001		< 0.001		< 0.001		< 0.001
No	116 (18.2)		75 (10.9)		56 (9.3)		14 (1.5)	
Yes	1148 (51.1)		978 (43.3)		523 (23.4)		223 (10.2)	

Estimates presented are unweighted numbers (weighted percent), and chi square *p* values

*Hurt wife/partner when she was not hurting the male respondent

**Table 3:** Univariate models (Unadjusted Odds Ratios) of males who report experiencing intimate partner violence in Uganda (2016)

Variable	All IPV	95%CI	*p*	Psychological	95%CI	*p*	Physical	95%CI	*p*	Sexual	95%CI	*p*
*Age*												
15−24	1			1			1			1		
25−34	0.99	0.73, 1.33	0.943	1.23	0.89, 1.69	0.210	1.04	0.69, 1.57	0.844	0.79	0.50, 1.25	0.319
35−44	1.12	0.82, 1.53	0.472	1.51	1.10, 2.06	0.010	0.96	0.63, 1.46	0.852	0.54	0.33, 0.88	0.014
45−54	0.93	0.66, 1.31	0.662	1.07	0.75, 1.51	0.724	1.27	0.81, 1.98	0.298	0.55	0.32, 0.96	0.035
*Residence*												
Rural	1.04	0.81, 1.34	0.763	0.96	0.76, 1.23	0.847	1.15	0.85, 1.56	0.361	0.88	0.58, 1.33	0.546
Urban	1			1			1			1		
*Education*												
No education	1			1			1			1		
Primary	1.28	0.77, 2.12	0.340	1.17	0.71, 1.92	0.536	0.99	0.59, 1.69	0.974	1.34	0.62, 2.87	0.453
Secondary	1.07	0.63, 1.83	0.803	0.90	0.53, 1.54	0.708	0.71	0.40, 1.24	0.227	1.48	0.66, 3.29	0.341
Higher	1.00	0.56, 1.79	0.997	1.00	0.56, 1.79	0.999	0.63	0.34, 1.16	0.140	0.85	0.36, 2.03	0.718
*Marital status*												
Married/ union	1			1			1			1		
Separated/ divorced	2.33	1.65, 3.28	< 0.001	2.53	1.79, 3.59	< 0.001	2.22	1.54, 3.18	< 0.001	2.04	1.30, 3.20	0.002
*Employment*												
No	1			1			1			1		
Yes	0.86	0.44, 1.67	0.653	1.47	0.74, 2.92	0.272	0.64	0.30, 1.34	0.236	0.99	0.33, 2.97	0.983
*Wealth index*												
Poorest	1			1			1			1		
Poorer	1.14	0.87, 1.50	0.337	1.01	0.77, 1.32	0.949	1.37	0.98, 1.92	0.061	2.12	1.23, 3.66	0.007
Middle	1.21	0.91, 1.61	0.185	1.20	0.89, 1.60	0.229	1.18	0.84, 1.65	0.348	1.74	1.00, 3.00	0.049
Richer	1.17	0.87, 1.57	0.309	1.15	0.84, 1.56	0.379	1.17	0.82, 1.67	0.400	1.86	1.07, 3.23	0.028
Richest	1.01	0.73, 1.39	0.955	1.02	0.74, 1.42	0.891	0.95	0.44, 1.41	0.792	1.80	1.00, 3.23	0.048
*No. children*												
0	1			1			1			1		
1	0.99	0.67, 1.48	0.978	1.37	0.85, 2.21	0.195	0.75	0.43, 1.31	0.318	0.94	0.49, 1.81	0.851
5+	1.09	0.73, 1.64	0.674	1.44	0.90, 2.31	0.132	0.93	0.53, 1.61	0.785	0.69	0.36, 1.34	0.275
*Smoking status*												
Does not smoke	1			1			1			1		
Some days	1.64	0.95, 2.83	0.074	1.63	0.95, 2.81	0.078	2.10	1.07, 4.09	0.030	0.83	0.38, 1.82	0.634
Everyday	2.10	1.57, 2.82	< 0.001	1.94	1.46, 2.57	< 0.001	2.04	1.49, 2.79	< 0.001	1.40	0.88, 2.24	0.158
*No. of wives*												
0 (Divorced/Separated)	1			1			1			1		
1	0.42	0.30, 0.59	< 0.001	0.39	0.27, 0.55	< 0.001	0.43	0.30, 0.62	< 0.001	0.50	0.32, 0.79	0.003
2+	0.50	0.33, 0.74	0.001	0.45	0.30, 0.68	< 0.001	0.56	0.35, 0.90	0.016	0.44	0.23, 0.83	0.012
*No. of sex partners*												
None	1			1			1			1		
One or more	2.17	1.74, 2.71	< 0.001	2.11	1.70, 2.62	< 0.001	1.88	1.47, 2.41	< 0.001	1.92	1.35, 2.74	< 0.001
*Given gifts for sex*												
No	1			1			1			1		
Yes	2.58	1.67, 4.00	< 0.001	2.34	1.55, 3.52	< 0.001	2.60	1.67, 4.05	< 0.001	2.73	1.64, 4.55	< 0.001
*Afraid of wife*												
Never	1			1			1			1		
Sometimes	3.15	2.44, 4.08	< 0.001	2.54	2.00, 3.24	< 0.001	3.16	2.41, 4.14	< 0.001	2.56	1.80, 3.64	< 0.001
Most of the time	7.94	4.39, 14.37	< 0.001	5.89	3.21, 10.80	< 0.001	7.37	4.24, 12.79	< 0.001	4.62	2.45, 8.71	< 0.001
*Father beat mother*												
No	1			1			1			1		
Yes	1.64	1.34, 2.00	< 0.001	1.57	1.29, 1.92	< 0.001	1.45	1.15, 1.84	0.002	1.29	0.89, 1.85	0.174
Don’t know	1.63	1.09, 2.43	0.018	1.63	1.08, 2.46	0.020	2.05	1.32, 3.18	0.001	1.12	0.58, 2.17	0.739
*Wife drinks alcohol*												
No	1			1			1			1		
Yes	2.58	2.03, 3.28	< 0.001	2.51	1.99, 3.18	< 0.001	3.10	2.41, 4.00	< 0.001	1.12	0.76, 1.65	0.581
*Frequency of wife being drunk*												
Never	1			1			1			1		
Sometimes	1.67	1.07, 2.62	0.025	1.26	0.80, 1.98	0.323	3.00	1.71, 5.26	< 0.001	3.39	1.24, 9.32	0.018
Often	3.76	1.59, 8.90	0.003	2.08	0.94, 4.61	0.070	6.31	3.00, 13.28	< 0.001	6.09	1.99, 18.63	0.002
Bi-directional violence*												
No	1			1			1			1		
Yes	4.39	3.47, 5.56	< 0.001	4.10	3.25, 5.16	< 0.001	4.00	3.19, 5.03	< 0.001	2.28	1.60, 3.26	< 0.001
*Controlling behaviour*												
No	1			1			1			1		
Yes	4.76	3.59, 6.29	< 0.001	6.26	4.57, 8.59	< 0.001	2.98	2.04, 4.34	< 0.001	7.53	4.20, 13.49	< 0.001

*Hurt wife/partner when she was not hurting the male respondent

*p* = *p* value

95%CI = 95% Confidence Intervals

**Table 4:** Multivariable model (Adjusted Odds Ratios) of males who reported experiencing intimate partner violence in Uganda (2016)

Variable	All IPV	95%CI	*p*	Psychological	95%CI	*p*	Physical	95%CI	*p*	Sexual	95%CI	*p*
*Afraid of wife*												
Never	1			1			1			1		
Sometimes	2.69	2.05, 3.52	< 0.001	2.12	1.64, 2.72	< 0.001	2.63	2.00, 3.47	< 0.001	2.10	1.46, 3.02	< 0.001
Most of the time	5.10	2.91, 8,96	< 0.001	4.28	2.50, 7.31	< 0.001	4.99	2.91, 8.53	< 0.001	3.59	1.87, 6.91	< 0.001
Bi-directional violence*												
No	1			1			1			1		
Yes	3.20	2.49, 4.12	< 0.001	2.99	2.33, 3.84	< 0.001	2.99	2.36, 3.78	< 0.001	1.87	1.29, 2.71	0.001
*Controlling behaviour*												
No	1			1			1			1		
Yes	3.80	2.84, 5.07	< 0.001	4.93	3.57, 6.81	< 0.001	2.03	1.38, 2.98	< 0.001	5.73	3.14, 10.46	< 0.001
*Father beat mother*												
No	1			1			1					
Yes	1.33	1.06, 1.66	0.013	1.28	1.02, 1.60	0.032	1.13	0.87, 1.47	0.350		NA	
Don’t know	1.41	0.92, 2.17	0.116	1.46	0.94, 2.25	0.092	1.88	1.17, 3.02	0.009			
*Wife drinks alcohol*												
No	1			1			1					
Yes	1.85	1.40, 2.45	< 0.001	1.83	1.39, 2.41	< 0.001	2.29	1.74, 3.01	< 0.001		NA	
*Number of sex partners*												
None	1			1			1					
One or more	1.42	1.11, 1.82	0.004	1.41	1.10, 1.81	0.007	1.25	0.95, 1.64	0.108		NA	
*Marital status*												
Currently married/union	1			1			1					
Separated/ divorced	1.61	1.09, 2.38	0.017	1.94	1.33, 2.82	0.001	1.57	1.07, 2.26	0.025	1.64	0.98, 2.74	0.058
*Age*												
15–24				1						1		
25–34		NA		1.37	0.98, 1.92	0.066		NA		0.77	0.46, 1.29	0.322
35–44				1.77	1.26, 2.47	0.001				0.52	0.30, 0.90	0.019
45–54				1.26	0.86, 1.85	0.241				0.56	0.31, 1.03	0.061
*Smoking*												
Does not smoke	1											
Some days	1.31	0.64, 2.65	0.432		NA			NA			NA	
Everyday	1.50	1.09, 2.06	0.014									
*Employment*												
No				1								
Yes	NA			2.70	1.29, 5.66	0.008		NA			NA	
*Wealth index*												
Poorest	1						1			1		
Poorer	1.25	0.94, 1.67	0.129		NA		1.47	1.02, 2.12	0.038	2.26	1.32, 3.89	0.003
Middle	1.43	1.05, 1.96	0.025				1.25	0.86, 1.82	0.236	1.84	1.05, 3.21	0.032
Richer	1.29	0.93, 1.80	0.129				1.19	0.81, 1.74	0.368	1.85	1.03, 3.30	0.039
Richest	1.18	0.83, 1.69	0.347				1.10	0.72, 1.66	0.664	2.15	1.19, 3.88	0.011

*Hurt wife/partner when she was not hurting the male respondent

*p* = *p* value

95%CI = 95% Confidence Intervals

NA represents not applicable for example, the variables not included in the final multivariable model

## Supplementary Information

Below is the link to the electronic supplementary material.

Supplementary file 1 (pdf 65 kb)
