# Supplementary material for: Ugandan Men Exposed to Intimate Partner Violence: A Cross-Sectional Survey of Nationally Representative Data
Source: J Prev (2022). 2022 Jun 1;43(4):567–88. doi: 10.1007/s10935-022-00683-2 (PMC9252969; doi:10.1007/s10935-022-00683-2)
Supplement: Supplementary file 1 — (pdf 65 kb) [file 10935_2022_683_MOESM1_ESM.pdf]

Table S1: Variables derivation from the Uganda, DHS data 2016

Variable name	Question	Code
IPV (all types)	A combination of either psychological, physical and sexual violence	Never (0); Yes (1)
Psychological violence	(Did your (last) (wife/partner) ever...):	
	Say or do something to humiliate you in front of others?	Never (0); Often/ Sometimes/ Yes, but not in the last 12 months (1)
	Threaten to hurt or harm you or someone you care about?	Never (0); Often/ Sometimes/ Yes, but not in the last 12 months (1)
	Insult you or make you feel bad about yourself?	Never (0); Often/ Sometimes/ Yes, but not in the last 12 months (1)
Physical violence	(Did your (last) (wife/partner) ever do any of the following things to you...):	
	Push you, shake you, or throw something at you?	Never (0); Often/ Sometimes/ Yes, but not in the last 12 months (1)
	Punch you with her fist or with something that could hurt you?	Never (0); Often/ Sometimes/ Yes, but not in the last 12 months (1)
	Kick you, drag you, or beat you up?	Never (0); Often/ Sometimes/ Yes, but not in the last 12 months (1)
	Try to choke you or burn you on purpose?	Never (0); Often/ Sometimes/ Yes, but not in the last 12 months (1)
	Twist your arm or pull your hair?	Never (0); Often/ Sometimes/ Yes, but not in the last 12 months (1)
	Threaten or attack you with a knife, gun, or other weapon?	Never (0); Often/ Sometimes/ Yes, but not in the last 12 months (1)
Sexual violence	(Did your (last) (wife/partner) ever do any of the following things to you...):	
	Physically force you to have sexual intercourse with her when you did not want to?	Never (0); Often/ Sometimes/ Yes, but not in the last 12 months (1)
	Physically force you to perform any other sexual acts you did not want to?	Never (0); Often/ Sometimes/ Yes, but not in the last 12 months (1)
	Force you with threats or in any other way to perform sexual acts you did not want to?	Never (0); Often/ Sometimes/ Yes, but not in the last 12 months (1)

Continued on next page

Table S1 – continued from previous page

Variable name	Question	Code
Age	How old were you at your last birthday?	15-24 (1); 25-34 (2); 35-44 (3); 45-54 (4)
Place of residence	Type of place of residence	Urban (1); Rural (2)
Level of education	What is the highest level of school you attended: primary, "O" level, "A" level, tertiary or university?	No education (0); Primary (1); Secondary (2); Tertiary (3)
Marital status	Are you currently married or living together with a woman as if married?	Currently in union/living with a woman (1); Formerly in union/living with a woman (2)
Working status	Have you done any work in the last 12 months?	No (0); Yes (1)
Wealth index	Wealth index combined	Poorest (1); Poorer (2); Middle (3); Richer (4); Richest (5)
Total number of children	Have you ever fathered any children with any woman?	No children (1); How many sons and daughters live with you? 1-4 children (2); And how many sons and daughters are alive but do not live with you? 5+ children (3); How many boys/girls have died
Smoking	Do you currently smoke tobacco every day, some days, or not at all?	Does not smoke (0); Every day (1); Some days (2)
Number of wives	Altogether, how many wives or live-in partners do you have?	No wife (0); One wife (1); Two or more wives (2)
Number of sex partners (12 months)	In total, with how many different people have you had sexual intercourse in the last 12 months, (excluding partner)?	None (0); One or more (1)
Given gifts for sex In the past 12 months	Have you given any gifts or other goods in order to have sex or to become sexually involved with anyone?	No (0); Yes (1)
Respondent afraid of wife	Are (Were) you afraid of your (last) (wife/partner): most of the time, sometimes, or never?	Never afraid (0); Most of the time afraid (1); Sometimes afraid
Father beat mother	As far as you know, did your father or any other husband or boyfriend your mother had ever hit or beat her?	No (0); Yes (1); Don't know (8)
Wife drinks alcohol	Does (did) your (last) (wife/partner) drink alcohol?	No (0); Yes
Frequency of wife being drunk	How often does (did) she get drunk: often, only sometimes, or never?	Never (0); Often (1); Sometimes (2)
Bi-directional violence	Have you ever hit, slapped, kicked, or done anything else to physically hurt your (last) (wife/partner) at times when she was not already beating or physically hurting you?	No (0); Yes (1)

Continued on next page

Table S1 – continued from previous page

Variable name	Question	Code
Controlling behaviour	Please tell me if these apply to your relationship with your (last) (wife/partner)? (She (is/was) jealous or angry if you (talk/talked) to other women?)	No (0); Yes
	She frequently (accuses/ accused) you of being unfaithful?	No (0); Yes (1)
	She (does/did) not permit you to meet your male friends?	No (0); Yes (1)
	She (tries/tried) to limit your contact with your family?	No (0); Yes (1)
	She (insists/insisted) on knowing where you (are/were) at all times?	No (0); Yes (1)
